# Progress in Medicine

**Published:** 1896-04-18

**Authors:** 


### Progress in Medicine.

### RENAL MEDICINE.

The diagnosis of interstitial nephritis in the earlier stages is one of the most difficult problems we meet with. The editor of the *Medical Record*[1] points out that we may be able to detect nothing beyond mental depression, dyspepsia, weakness, and rheumatic pains. Albumen may be absent for a long period, the specific gravity of the water may rise as the heart failure increases, and except for an occasional hyaline cast there may indeed be nothing abnormal in the urine. This indefinite malaise, with even a daily rise of temperature, may go on for years before decisive symptoms appear. Ralph noticed that he had often been led to a correct diagnosis in these cases by the presence of persistent neuralgic pains. The frequent or periodic appearance of casts in non-albuminous urine is discussed in a paper by F. Bremer,[2] who insists that they are a sign of diseased or at least of abnormally delicate kidneys. Though they have been noticed in the water of well-known athletes after exertion, he would regard them as a sign of a damaged constitution.

Kossler[3] found casts in the water of a majority of twenty-nine patients who were suffering from various diseases, and none of whom had albuminuria. Tubercular patients are specially liable to pass these casts. In many of his cases Kossler found no evidence post-mortem of nephritis, but only of patches here and there of degenerative changes in the epithelia of the tubules.

The various causes of dysuria have been discussed in a recent lecture by C. H. Ralfe,[4] both those dependent on changes in the urine or in some part of the urinary tract, and those which are the result of reflex neuroses. Among other details he pointed out that occasionally in paroxysmal hæmoglobinuria there is dysuria indistinguishable from that of vesical calculus. Again, the pain in oxaluria is felt chiefly in the perineum, that from uric acid all along the urethra, while that from phosphates is chiefly at the neck of the bladder and at the end of micturition. It is interesting to find such an authority as Dr. Ord[5] recommending as a practical test for albumen the addition of $HNO_3$ (1 in 4), or glacial acetic acid (2 in 4) after boiling, while in using picric acid for the same purpose he first boils the urine, then adds an excess of picric acid, and then boils again. The first boiling eliminates phosphates, and without the second we may wait some time before precipitation of albumen occurs, or may mistake a cloud caused by quinine, uric acid, or peptones. Though mucin or nucleo-albumen under either of these tests gives a slight precipitate, it can easily be distinguished by "the satiny lines of light which follow all movements of the fluid in the shaken tube," where it is present.

E. E. Smith[6] recommends that urine should be filtered perfectly clear, a few drops of acetic acid or liq. potassæ being first added when necessary for this purpose. Afterwards the results of the ferrocyanide or heat with nitric acid tests should be checked by a comparison tube to which a little acetic acid has been added. Slight cloudiness in them will be due to nucleo-albumen if this tube shows it too. A rapid method of testing for albumen by asaprol is described by Riegler.[7] This substance precipitates albumen in acid solutions; the precipitate is soluble in potash, and the refractive index of the solution is in direct relation to the amount of albumen present, and is easily determined by Pulfrich's refractometer. Truax[8] advocates the use of alcohol of 60 to 80 per cent. strength instead of the common tests. When urine is dropped into it white streaks are seen if albumen is present, but if only mucus is there, a diffused cloud is seen. The test may be also varied by floating the alcohol on to the surface of the urine. Nicolaer[9] has lately pointed out the remarkable properties of urotropine, an amine compound of formalin. He found that it is a powerful diuretic, as well as a solvent of uric acid, and an antiseptic for the urinary tract. Putrid and alkaline urine or that containing ropy mucus on the one hand or uric acid crystals or calculi on the other is rapidly rendered healthy by its administration. It combines well with salicylic acid, ten or fifteen grains of both drugs together being soluble in an ounce of water. Though not poisonous even in much larger doses, he recommends a daily dose of from eight to twenty grains of urotopine as sufficient for ordinary purposes. Investigations on the metabolism and excretion of caffeine and theobromine[10] show that much of the alkaloid is excreted as monomethyl-xanthine, and that the amount excreted and the diuresis provoked varies with different animals. A. Foxwell[11] has recorded two cases of uræmia in which cold water enemata were employed. In one instance rapid improvement, physical and mental, followed; in the other, where the power of reaction was absent, the œdema was increased, and the end seemed to come sooner in consequence. The length of life in albuminuric retinitis has been estimated by several authorities as under two years. This has been studied by Possaner[12] in seventy-two cases. Twenty-seven out of thirty-three hospital patients thus affected died in two years, but one at least survived six years. Among private patients nearly half survived the limit, and one reached eleven years. The prognostic importance of traces of albumen, according to Lionel Beale,[13] can only be estimated rightly by prolonged watching for several months. Every trace of kidney disease, even when casts have been present, will sometimes disappear, especially in the young, and even when it occurs in men above forty it does not necessarily shorten life. On the other hand, the renal disease may become rapidly progressive, or any acute malady may be fatal in albuminuric patients. Among other sources, the albumen may come from a small calculus, a villous growth, or from a minute area of disease in the kidney. Reginald Harrison,[14] in discussing those curious cases which have recovered entirely after an exploratory operation for supposed calculi, suggests that much albuminuria is due to inflammatory changes in the kidneys, with accompanying tension in the gland, and that where, after acute nephritis, the albumen does not tend to disappear, an incision through the kidney substance offers a possible chance of relief, with little or no risk. Memminger and Evans[15] deny that albuminuria is primarily a disease of the kidneys, and consider it an affection of the cells of the entire body which produces rejection by the cells of albumen. The kidney suffers not only from the disease of its own cells, but also from the stress of the additional elimination thrown on it from the general cell irritation. The theory is not altogether unlike that of Semmola. Another method of detecting minute amounts of albumen may be mentioned here, which has just been described by Jolles.[16] Ten grammes each of common salt and chloride of mercury, with 20 of succinic acid, are dissolved in 500 grammes of distilled water. To 4 c.c. of the filtered urine 1 c.c. of acetic acid is added, and then shaken up with 4 c.c. of this reagent. A cloudiness is produced by extremely minute traces of albumen. For comparison Jolles adds similar quantities of acetic acid and water to urine in a second tube. Oxaluria was the subject of much careful work by J. C. Dunlop,[17] which was discussed at the Edinburgh Medical Chirurgical Society. Dunlop found that the urine of all persons eating an ordinary mixed diet contained oxalic acid, but that the dumb-bell crystals commonly believed to be oxalates were really carbonate of lime. The cause of precipitation seemed to be chiefly from an excess of oxalic acid, which, in turn, was derived from that contained in the food. It was not formed by any metabolism within the body. The symptoms of Begbie's oxaluria were simply those of acid dyspepsia, except that the lumbar pain might be due to the irritation caused by the passage of the crystals. The presence of oxalates in the urine was of no importance, and was not a sign of disease. It could be put an end to by removing all oxalates from the food, and produced by giving substances such as tea which contain it. Stockman and others pointed out that oxalates might have other sources in certain cases of disease, besides those which Dunlop proved to exist in health. In discussing the production of uræmia, T. Oliver[18] remarks that we have a group of symptoms which are seen, not only in cases of Bright's disease, but also in certain others of dilated stomach, diabetes, lead poisoning, liver disease, and pregnancy. The lessened elimination of urea which precedes many attacks does not explain the pathology. There is a retention, however, of some poisonous matters in all these cases, and even the urine in puerperal eclampsia is less toxic than normal. Sometimes there is direct absorption of a poison from the gastro-intestinal tract with insufficient elimination. Diuretin appears somewhat uncertain in its action in nephritis, though, according to Askanazy,[19] very valuable in cardiac dropsy. He finds that doses of 45 to 60 grains a day should not be exceeded. The use of the centrifuge for the estimating the significance of slight albuminuria is pointed out by Zeehuisen,[20] as by its means the presence or absence of casts, blood corpuscles, bacteria, &c., is readily detected, and the true importance of the albumen can thus be estimated. The presence of bacteria too in the urine does not necessarily imply disease of the urinary tract, for as Biddell and Krauss showed, staphylococci, bacillus coli, anthrax, and others when injected into the blood stream rapidly appear in the urine. Thus the kidneys are easily pervious to bacteria contained in the general blood-stream of the body.

1 Med. Rec., Dec. 7. 2 Med. Rec., Dec. 28. 3 Lanc., Dec. 14. 4 Int. Clinics, iv., 2. 5 Pract., Jan., 1896. 6 Med. Rec., Jan., 4. 7 B.M.J., Dec. 21. 8 N.Y. Med. Jour., Nov. 2. 9 Therapist, Mar. 16. 10 Med. Chronic., Nov. 11 Birming. M. Rec., Nov. 12 B.M.J., Jan. 25. 13 Internat. Clinics, iv.. 3. 14 Lanc., Jan. 4. 15 Am. M. and S. Bulletin, Jan. 11. 16 Lanc., Feb. 17 Edinb. Med. J., Jan. 7. 18 Int. Clinic, iv., 4. 19 Practit., March. 20 Practit., March.

